# Insulating Material with Scale Components for High-Temperature and High-Pressure Water Applications

**DOI:** 10.3390/molecules29174046

**Published:** 2024-08-27

**Authors:** Xiaoqiang Zhao, Zongyong Lou, Yide Gao, Wenhui Feng, Dong Wang, Xiao He

**Affiliations:** 1 Department of Thermal Engineering, Hebei Petroleum University of Technology, Chengde 067000, China; zxq130823@163.com (X.Z.); louzongyong112@126.com (Z.L.); gaoyd106@163.com (Y.G.); wenhuicuihua@126.com (W.F.); 2 School of Energy and Power Engineering, Xi’an Jiaotong University, Xi’an 710049, China; wangdong@mail.xjtu.edu.cn

**Keywords:** scale components, glass glaze, composite glaze, firing, high temperature and high pressure in water, insulating materials

## Abstract

Accurately measuring water holdup in horizontal wells is crucial for effectively using heavy oil reservoirs. The capacitance method is among the most widely used and accurate techniques. However, the absence of suitable insulating materials at high temperatures and pressures limits the effectiveness of capacitive water holdup measurement in heavy oil thermal recovery. This study introduces a new composite material based on an aviation-grade, special glass glaze as the insulating medium doped with inorganic components (CaSO_4_, MgSO_4_, Ca(OH)_2_, and SiO_2_). This new composite material demonstrates outstanding insulating performance under high-temperature and high-pressure conditions in water. A water environment with a high temperature of 350 °C and a pressure of 12 MPa considerably enhances the composite material’s insulation. After 72 h of continuous use, the insulation performance remains 0.3 MΩ. The layers exhibit improved insulation and stability, maintaining integrity through five consecutive temperature shocks in 500 °C air and 20 °C water. XRD, IR, SEM, and TEM analyses reveal that the new composite material is amorphous after firing and that the addition of inorganic components improves the bonding between the glass glaze components and contributes to a denser structure. Simultaneously, SEM and TEM analyses indicate that adding inorganic components results in a smoother, crack-free, and more compact surface of the special glass glaze. This enhancement is crucial for the material’s long-term stability in high-temperature and high-pressure water environments.

## 1. Introduction

Heavy oil thermal recovery using horizontal well technology has become an important research area. Water holdup measurement is a core indicator of this technology [1,2,3,4,5]. Current methods for measuring water holdup include radiation, differential pressure, orifice throttling, and optical methods [6,7,8,9,10]. However, these methods cannot achieve the real-time measurement of water holdup in the confined space of a horizontal well at temperatures above 300 °C and pressures exceeding 10 MPa. The prediction method and sampling test currently measure water holdup in horizontal wells, but they have poor accuracy, stringent conditions, and complex operation. The capacitance method for measuring downhole water holdup is preferable for its accuracy and simplicity [11,12,13]. This method requires the substrate to be a good conductor, with a thin-walled insulating layer tightly applied to the outside. The insulating material must exhibit excellent insulation properties, high-temperature and pressure resistance, and minimal impact from water surface tension after immersion. However, finding an insulating material that remains stable over long periods under the downhole conditions of variable temperature and pressure in a water environment has become a considerable challenge in developing this technology.

Scholars primarily focus on the insulation performance of materials in air [14,15,16,17,18,19]. Chen et al. [17] fabricated a nanocomposite film by vertically folding polymer nanocomposite fibers using electrospinning and hot pressing. This film exhibits excellent electrical insulating properties and thermal conductivity (16.3 W m^−1^ K^−1^) at room temperature and normal pressure in air. Zhang et al. [15] successfully developed polyimide/polyvinyl alcohol composites with thermal conductivity and electrical insulation properties. As a result of doping with 0.3% carbon nanotubes and 30% boron nitride nanosheets, the composite achieved good flexibility, acid and alkali resistance, and electrical insulation properties. Despite the effectiveness of these insulating materials at room temperature and normal pressure, research on the insulation performance of materials in water under variable temperature and pressure conditions remains limited.

Insulating enamel circuit substrate materials are widely used in various industries, including communications, automobiles, national defense, cameras, space technology, and medicine, due to their low cost, high-temperature resistance, stable performance, and high mechanical strength [20,21,22]. However, these applications are mainly concentrated in nonliquid media, with fewer uses in insulation materials in high-temperature, high-pressure water environments.

In this paper, to ensure the material’s excellent insulation performance at high temperatures and pressures in water, a layer of special glass glaze is first fired as the base layer on 316L stainless steel. The special glass glaze is added to CaSO_4_, MgSO_4_, Ca(OH)_2_, and SiO_2_ to enhance its performance in water further. After grinding and screening, the mixture is sintered as a top glaze layer. The surface exhibits a mirror-like gloss once sintered and naturally cooled to room temperature. When fired onto 316L stainless steel parts, this glass glaze demonstrates high-temperature resistance, aging resistance, and excellent insulation properties. In a water environment with a temperature of 350 °C and a pressure of 12 MPa, the insulation performance remains effective after continuous use for 72 h, with resistance still at the megaohm level (0.3 MΩ). Additionally, the material can withstand sudden temperature changes between 500 °C air and 20 °C water for five consecutive cycles. Verification against the actual conditions and requirements of heavy oil thermal recovery sites has confirmed that the material fully meets the necessary standards for on-site use. Currently, the material has been practically applied in these environments.

## 2. Experimental Procedures and Discussion

### 2.1. Experimental Instruments and Medicines

Main equipment for the experiment: A BJMC-TM0413 muffle furnace (Beijing Ying’an Mei cheng Scientific Instrument Co., Ltd., Beijing, China) (operating at 1300 °C), a lathe (Shenyang Machine Tool Factory, Shenyan, China), a Fluke PM6304 capacitance measuring instrument (Fluke International Corporation, Washington, DC, USA), a HEWLETT-PACKARD 34,401 multimeter (Hewlett-Packard Development Company, Palo Alto, CA, USA)and a FLUKE-F1503 multimeter (Fluke International Corporation, Washington, DC, USA), a piezometer (Zheng bao Instrument Co., Ltd., Shanghai, China) (0–30 MPa), high-pressure water-filled container made by ourselves, and a pressure adjustment valve Zhengbao Instrument Co., Ltd., (Shanghai China) (12 MPa),a PERTHOMETERS2 surface roughness tester (Mahr GmbH, Esslingen, Germany), a Mitutoyo 0–150 mm Vernier calipers (Mitutoyo, Kawasaki, Japan) a Bruker D8 diffractometer (BRUKER AXS GMBH, Berlin, Germany), a Bruker VERTEX 70 spectrophotometer (BRUKER AXS GMBH, Berlin, Germany), an FEI Tecnai G2 F20 S-TWIN microscope (BRUKER AXS GMBH, Berlin, Germany), super-high-resolution Hitachi SU8010 field emission microscope (BRUKER AXS GMBH, Berlin, Germany).

Materials: Special glass glaze, CaSO_4_, MgSO_4_, Ca(OH)_2_, SiO_2_, tap water, distilled water, and 316L stainless steel were used. Stainless steel was prepared using 1500-grit sandpaper at 1250 rpm and cleaned with acetone and other fluids. All sintered parts had circular-arc transitions on edges and corners.

Roughness measurement: We prepared the 316L stainless steel sample using a lathe the lathe speed was set to 1250 rpm. Then, the stainless steel base was ground using the 1500-grit sandpaper. Finally, the surface roughness was measured using the PERTHOMETERS2 surface roughness tester. 

Composite-glaze-thickness testing: After processing the 316L stainless steel base, we measured its thickness 20 times along the radial and axial directions using Mitutoyo 0–150 mm Vernier calipers and calculated the average value A. Once the composite glaze was fired on the substrate, we measured the thickness again 20 times in both directions using the same calipers to obtain the average value B. The thickness of the fired composite glaze was then calculated as (B − A)/2.

Resistance testing: During resistance measurement, two wires were drawn out from the outer wall and body of the test piece. Then, the resistance was measured using a multimeter and the capacitance was measured using a PM6304 capacitance measuring instrument. All measurements were performed in triplicate.

Measurements and characterization: X-ray diffraction (XRD) patterns were obtained using a D8 diffractometer with a CuKα radiation source operating at 40 kV and 100 mA with a step size of 0.01 1/4 2q in the range of 10–80°. Fourier transform infrared (FT-IR) spectra were recorded using a VERTEX 70 spectrophotometer using KBr pellets. Transmission electron microscopy (TEM) images were obtained using an FEI Tecnai G2 F20 S-TWIN microscope at an extraction voltage of 3950 V, gun lens of 1, and spot size of 1. SEM images were acquired using the super-high-resolution Hitachi field emission microscope with an EDX spectrometer.

### 2.2. Experimental Section

#### 2.2.1. Sintering and Testing of Special Glass Glaze Materials (Specimen ①)

The bottom glaze uses special glass as the primary material, with its main components listed in Table 1. Before firing, the special glass glaze material is ground and sieved using a 260-mesh stainless steel sieve. It is then mixed with distilled water at a mass ratio of 3:2 (with viscosity adjusted as needed). The prepared liquid slurry is filtered through a 600-mesh sieve before use.

**Figure 1:**
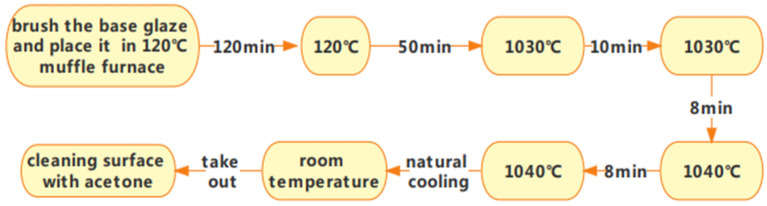
Flowchart of the glass-glaze firing process.

After substrate treatment, it is evenly dipped and coated with a uniform mixture of special glass and distilled water. Once coated, the substrate is rapidly heated in a muffle furnace at 120–180 °C. Temperatures above 180 °C may cause the water to evaporate too quickly, compromising the sintering quality. After sintering and molding, the substrate can cool naturally to room temperature. Figure 1 illustrates the sintering process.

**Figure 2:**
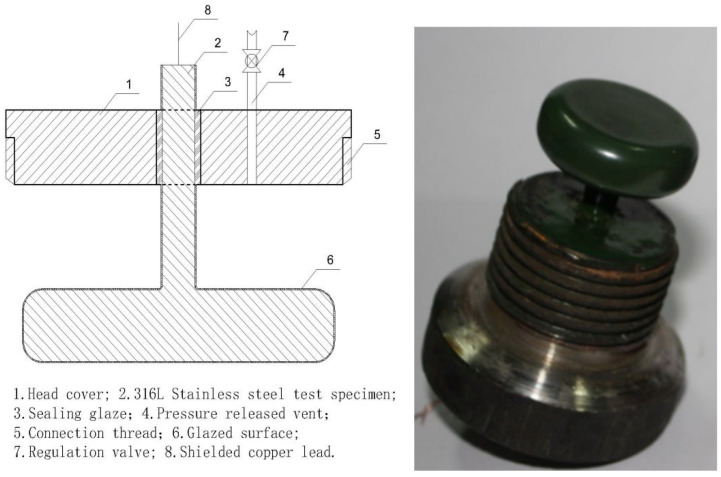
Cross-section of the sintered structure and connecting structure of the top cover and forming sample.

The sintering process was performed twice according to the molding steps outlined above (Figure 1) to ensure the comprehensive quality and performance of the glaze surface. Additionally, because insulation testing in high-pressure water was required, the sintered glazed structural parts needed to be isolated from the water, requiring a threaded top cover and a test container. Specifically, a hole was made in the center of the top cover (with a diameter equal to the sintered structure’s shank diameter plus 2 mm). The sintered structure was then inserted into the top cover, ensuring concentric alignment, and the hole was filled with glaze before final sintering. The sintering steps follow the same procedure as described above. It is important to maintain a molding temperature of 1050 °C and hold it for 15 min to ensure the full fusion of the glaze during both the first and second sintering stages. Figure 2 illustrates the schematic and molding diagrams showing the connection between the top cover and the base after sintering.

#### 2.2.2. Sintering and Testing of Prepared Glass Glaze (Specimen ②)

To extend the glaze’s service life under high temperature and pressure in water, CaSO_4_, MgSO_4_, Ca(OH)_2_, and SiO_2_ are added to the dry powder of the special glass glaze for use as the surface glaze. Table 2 provides the mass ratios.

**Figure 3:**
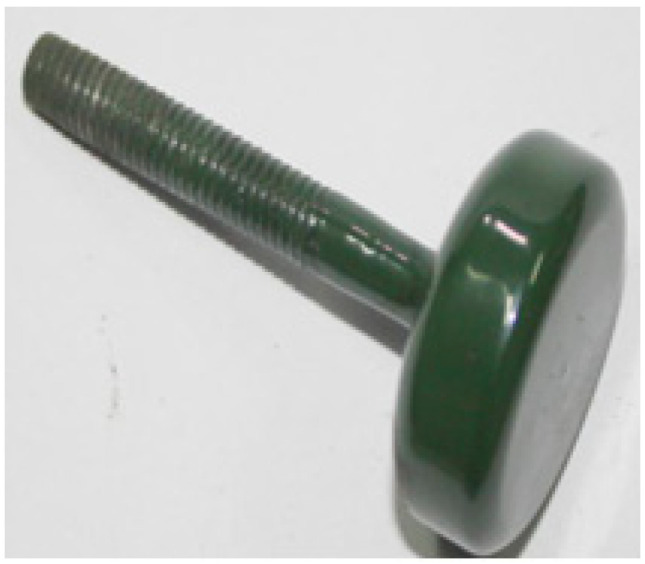
Sintering diagram of special glass glaze material specimen ②.

**Figure 4:**
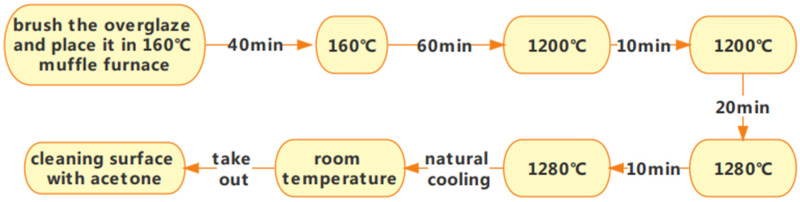
Preparation process flow chart 2.

Grind the material for approximately 1 h before sintering the surface glaze, and then sieve it through a 420-mesh stainless steel sieve. Next, mix the powdered material with distilled water in a mass ratio of 7:5. Before use, filter the prepared liquid slurry through a 600-mesh sieve. After filtration, let the slurry stand for 2.5–3 h to allow clean water to separate. Stir the slurry thoroughly before brushing it onto the firing surface glaze. This process ensures the optimal sintering performance of the glaze and the stainless steel substrate. Before sintering the glaze material, a layer of special glass glaze (the bottom glaze) should be sintered on the substrate according to the first sintering process for the special glass glaze. Once the bottom glaze is sintered, apply and sinter the glass glaze as the top layer, following the specifications for the sintered specimen ①. The glass glaze is applied between the top cover and the first sintered structural component. The finished product prepared in this manner is labeled as a specimen ②. Figure 3 shows a smooth and flat specimen ②. Changes in material composition affect the temperature of this sintering process, as depicted in Figure 4.

#### 2.2.3. Sintering and Testing of Prepared Glass Glaze (Specimens ③, ④, and ⑤)

As shown in Table 3, Table 4 and Table 5, specimens ③, ④, and ⑤ were prepared precisely the same way as specimen ②, except for the composition.

### 2.3. Insulation Resistance Dependence on the Temperature and Time in Different Environment Conditions

**Figure 5:**
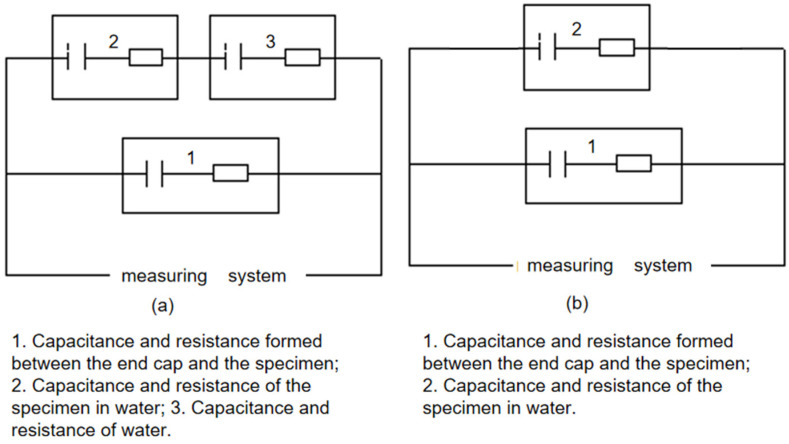
Specimen test equivalent circuit diagram ((**a**) Insulation medium measurement test equivalent circuit diagram; (**b**) Test equivalent circuit diagram with water as conductor).

The primary objective of the conducted experiment was to evaluate the insulation performance of specimens ①–⑤ in both air and water environments. Based on the principles of electrolytic capacitors, the experimental design utilized models where industrial water served as an effective conductor relative to the insulating dielectric. As depicted in Figure 2, the materials of elements 6 and 3 differ, thereby developing distinct capacitances and impedances with their respective electrolytes and forming two parallel capacitors. Moreover, while water functions as a conductor, it also exhibits its own capacitance and impedance. When substituted with air, which possesses limited conductivity, the presence of minimal capacitance and impedance is still observed. Thus, measurements in air were conducted using the same equipment, and results were directly obtained from the measuring meter. Consequently, the final test equivalent circuit was established (Figure 5a). Given that Liu et al. [23] explored water’s efficacy as a conductor and considering the negligible yang value produced by industrial water, a simplified equivalent circuit is presented in Figure 5b).

**Figure 6:**
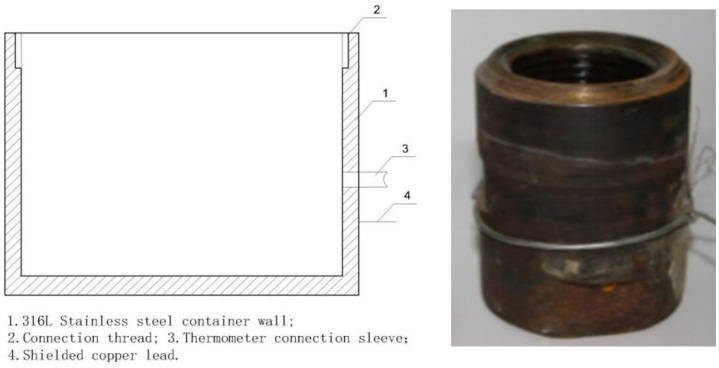
Schematic diagram and image of the test vessel.

**Figure 7:**
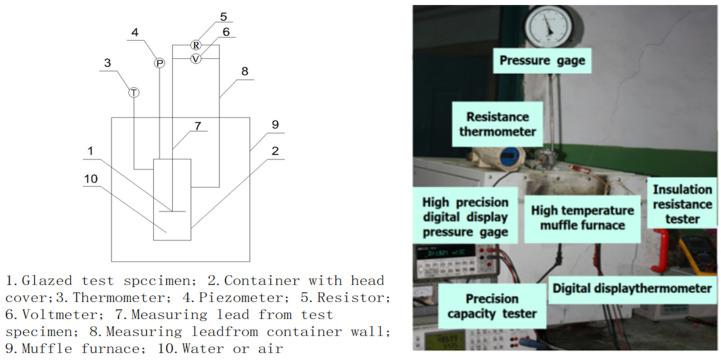
Test equipment composition and physical picture.

During testing, we modified an interface container with threading (Figure 6), which contained only air when measured in air and was filled with industrial water when measured in water. The test structure was assembled as shown in Figure 7, with the left side depicting the arrangement of test equipment and the right side displaying a physical photograph of the setup.

In the procedure, the object on the right side of Figure 2 was inserted into the corresponding container on the right side of Figure 6 and secured using the thread at room temperature, forming a unified assembly (see 1 and 2 in Figure 7). The connections involved copper core shielded signal wires, with wire 7 linked to element 1 and wire 8 to element 2. During measurements, elements 1 and 2 were placed in muffle furnace 9 following reinforcement, with their temperature meticulously adjusted. Once the temperature stabilized, data were immediately recorded.

When measurements are conducted in air, the temperature is recorded using digital thermometer 3, and the resistance is assessed with a multimeter 5 (FLUKE-F150 in air). The leads of multimeter 5 are connected to the ends of wires 7 and 8, which are secured with insulating tape before measuring resistance. Following data recording at this temperature, the system is heated progressively until the target temperature is achieved. Detailed descriptions of the measurement procedures and precautions can be found in Section 2.3.1, with the measurement results presented in Table 6.

In water-based measurements, the temperature is logged by digital thermometer 3, the pressure of saturated water by pressure gauge 4, and the resistance by multimeter 5. Although not discussed in this paper, a Fluke PM630 multimeter measures the potential difference for future engineering considerations. Similar to air measurements, the leads of multimeter 5 are connected to the ends of wires 7 and 8, with resistance measured post-securing by insulating tape. The process continues with heating until the desired temperature is attained. For further details on the measurement protocol and precautions, refer to Section 2.3.2 and Section 2.3.3, with corresponding results in Table 7 and Table 8.

**Figure 8:**
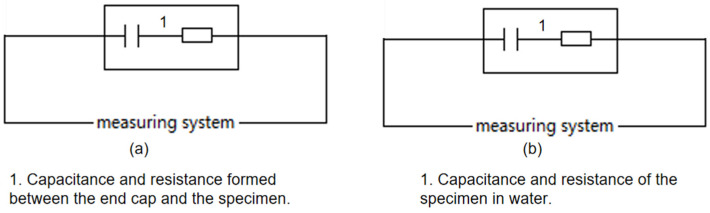
Simplified circuit diagram ((**a**) Test equivalent circuit diagram for measurement in air. (**b**) test equivalent circuit diagram for measurement in water).

According to Figure 6 and Figure 7, when measurements occur in air, the resistance primarily emerges between elements 1 and 2, as depicted on the left in Figure 2. This resistance, typically in the hundreds of MΩ range, indicates excellent insulation, as illustrated simplistically in Figure 8a. In water tests, although there is no direct contact with water due to the sealed interface between elements 1 and 2, the measured resistance mainly arises between elements 1 and 10 in Figure 7. This setup is intended to approximate the insulation performance of specimens ②–⑤ under high temperature and high-pressure water conditions, with a corresponding simplified circuit diagram presented in Figure 8b. The measurement data from these water tests provide an objective reflection of the insulation properties of the tested samples.

#### 2.3.1. Air Temperature Test

Connect the sintered specimen ① to the stainless steel container and place it in the muffle furnace, following the connection method outlined in the flow chart of the test principle. Before beginning the test, ensure that all measuring instruments are properly fixed, connected, and adjusted, and shield any potential interference signals. During the experiment, the muffle furnace temperature was adjusted as needed, and data were recorded each time the temperature increased by 30 °C and stabilized. Repeat this process three times to ensure accurate recording. The temperature change rate should not exceed 1.5 °C/10 min, and the overall temperature variation should not exceed 3 °C/30 min. Additionally, the temperature difference between the constant temperature of specimen ① and the set temperature of the muffle furnace should not exceed ±5 °C until reaching 450 °C. After the test, allow the muffle furnace to ventilate and cool naturally. The testing procedure for specimen ② is identical to that of specimen ① and will not be repeated here.

#### 2.3.2. Normal Temperature Water Test

Fill the stainless steel container with tap water and connect the sintered specimen ① to the container. Drain the excess water through the pressure regulating valve, and then place the container in the muffle furnace. Connect the meters according to the connection method detailed in the flow chart of the test principle, ensuring that the bottom part of the top cover is fully immersed in the water. Before the test, ensure that all measuring instruments are securely fixed, properly connected, adjusted, and shielded from interference signals. During the experiment, measure the data every 3 h over 72 h, with the water temperature maintained at 20 ± 5 °C. The testing procedure for specimen ② is identical to that of specimen ① and will not be repeated here.

#### 2.3.3. Test in Variable Temperature and Pressure

Fill the stainless steel container with tap water, install the test specimen ① in the container, and place it in the muffle furnace. Connect the pressure relief hole to a piezometer and install a water discharge capillary in the pipeline. Display the data using the piezometer and regulate the pressure with the pressure regulating valve, ensuring it does not exceed 12 MPa. Before the test, ensure all measuring instruments are securely fixed, connected, properly adjusted, and shielded from interference signals. During the experiment, adjust the muffle furnace temperature as needed. Record the data each time the temperature increases by 30 °C and stabilizes; three consecutive recordings are required. The temperature change rate should not exceed 1.0 °C per 10 min, and the overall temperature variation should not exceed 2 °C per 30 min. Additionally, the temperature difference between the constant temperature of specimen ① and the set temperature of the muffle furnace should not exceed ±5 °C until the temperature reaches 350 °C. After the test, allow the muffle furnace to ventilate and cool naturally.

The temperature adjustment requirements are consistent with those for temperature testing in air. The testing procedure for specimen ② is identical to that of specimen ① and will not be repeated here.

#### 2.3.4. Temperature Shock Test

After completing the temperature and pressure performance tests for specimen ②, remove and disassemble it. Then, place specimen ② into the muffle furnace, adjust the temperature to 500 °C, and maintain this temperature for at least 5 h. After this period, quickly remove specimen ② from the furnace, place it into a container with a diameter of 350 mm and a height of 400 mm, and fill the container with water at 20 °C. Upon immersion, a large number of bubbles will rapidly form on the surface of the specimen. Once stabilized, remove the test specimen and observe its surface condition, completing the sudden temperature change test. After this initial impact test, allow the specimen to cool naturally to room temperature. Then, place it back in the muffle furnace and heat it to 500°C for a second test. After five consecutive tests, examine the surface condition of the specimen and measure its insulation performance in tap water to check for any shedding.

## 3. Results and Discussion

### 3.1. The Insulation Resistance Dependence on the Temperature and Time in Different Environment Conditions of Composite Glaze in Air

Table 6 shows the performance data for specimen ① made of special glass glaze in air. The resistance is infinite at a temperature of 17–200 °C, indicating stable insulation. With increasing temperature, the resistance gradually decreases. At 448.3 °C, the resistance is 567.9 MΩ, which remains high (in the megaohm range), placing it between insulators and semiconductors and generally maintaining its insulating properties. However, during testing, the insulation performance considerably deteriorated after 20 min, with approximately 70% of the insulating layer peeling off upon removal. This may arise from ordinary enamel materials’ poor mechanical properties and adhesion and insulation characteristics. Doping with inorganic components effectively enhances composites’ insulation, mechanical properties, and adhesion [24,25,26,27]. For instance, Ota and Harada [27] incorporated magnesium oxide fillers into epoxy resins containing mesogens to fabricate high-thermal-conductivity epoxy composites. Their study demonstrated that magnesium oxide effectively promotes the self-assembly of highly ordered smectic crystals, considerably improving the material’s insulating properties, adhesion, stability, and mechanical performance. Thus, selecting appropriate fillers for doping into the polymer substrate and synchronizing the polymer substrate and filler orientation is crucial. The objective is to build on this approach and enhance material properties by incorporating inorganic components. Because scale is known for its excellent insulating properties, adding scale components to the enamel glaze may considerably improve the composite material’s insulation performance under high temperatures and pressure in water. Previous experiments revealed that, after natural air-drying, a thin layer of light white powder adhered uniformly to the 316L stainless steel body. This white powder proved stable in environments with variable temperature and pressure. Composition tests of the white powder identified strong components such as Ca(OH)_2_, Mg(OH)_2_, CaCO_3_, CaCl_2_, MgCl_2_, CaSO_4_, and MgSO_4_, which are the main components of scale.

Considering that the special glasses used in the previous tests have a melting point of approximately 1150 °C, adding scale-related components to these special glasses was investigated to assess their performance under variable temperature and pressure conditions in water. Specifically, CaCO_3_ decomposes at approximately 900 °C, while MgCO_3_ decomposes at approximately 350 °C. MgSO_4_ has a melting point of approximately 1200 °C, close to the test glass’s. CaSO_4_, a colorless monoclinic crystalline powder with a vitreous luster, has a melting point of 1450 °C for the monoclinic form and 1193 °C for the orthorhombic form. The melting point of both CaSO_4_ and CaCl_2_ is 770 °C, while MgCl_2_ has a melting point of 801 °C. Calcium carbonate and magnesium carbonate are unused due to their decomposition at lower temperatures, which ensures a smooth surface during special glass sintering and prevents gas formation. Additionally, MgSO_4_ has a melting point lower than 1150 °C, required for the sintering process. This lower melting point makes MgSO_4_ prone to falling off in the melt state, rendering it unsuitable for this application.

Ca(OH)_2_ melts at 580 °C, roughly in the middle range of the sintering temperature for the material used in this case. It can be effectively used in moderation as a preliminary molding agent. CaSO_4_ and MgSO_4_ are preferred choices for enhancing the scale components. Their melting points are close to that of the special glass, allowing for effective integration upon crystallization and incorporating a suitable amount of Ca(OH)_2_ aids in the molding process. Furthermore, when CaSO_4_ transitions to its monoclinic crystal form, it establishes a structural core that considerably stabilizes the structure and enhances the mechanical properties of the material sintered on the stainless steel body.

Additionally, it is crucial to incorporate wear-resistant insulating materials to address the harsh conditions encountered when the equipment is used underground to minimize wear and tear. Mixing appropriate amounts of SiO_2_ and CaSO_4_ and using the “crystal nucleus” effect enhances the material’s overall performance for sintering and testing. Accordingly, specimen ② was prepared by adding CaSO_4_, Ca(OH)_2_, MgSO_4_, and SiO_2_ to the special glass glaze. The test results for specimen ② were similar to those for specimen ①, further demonstrating the material’s excellent insulation performance in air and at high temperatures. Fortunately, specimen ② adhered well and did not experience any delamination.

### 3.2. Variation of Resistance with Temperature and Time Test of Composite Color Glaze in Water at Different Times

Table 7 presents the performance data for specimens ② and ① in normal-temperature water over various times after incorporating CaSO_4_, Ca(OH)_2_, MgSO_4_, and SiO_2_ into the colored composite glaze. The data in Table 6 show that the insulation resistance of specimen ①, fired with a special glass glaze without inorganic additives, decreased sharply from 98.5 to 5.5 MΩ during the test. This rapid change occurred because, initially, specimen ① was only partially wetted, leading to a higher insulation resistance because water molecules had not fully contacted the surface. The insulation resistance decreased quickly as the water molecules interacted more closely with the surface. As time progressed, the insulation resistance of test specimen ① gradually decreased, becoming comparable to that of a conductor after 30 h, indicating considerable insulation degradation. Upon removal, the specimen’s surface submerged in water lost its luster, turned dark green, and exhibited noticeable roughness and unevenness. Areas with a diameter of approximately 0.5 mm had detached, revealing the stainless steel substrate beneath. The water in the container had turned a light yellow-green color. Measurements showed a slight reduction in the glaze thickness, suggesting that the glaze material had dissolved in the water.

**Figure 9:**
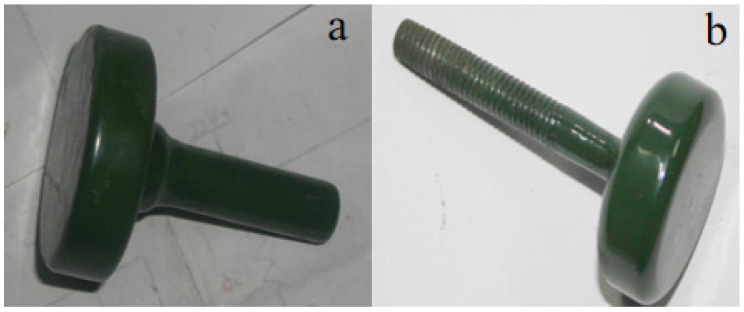
Color morphology of sample ② before (**b**) and after (**a**) testing.

**Figure 10:**
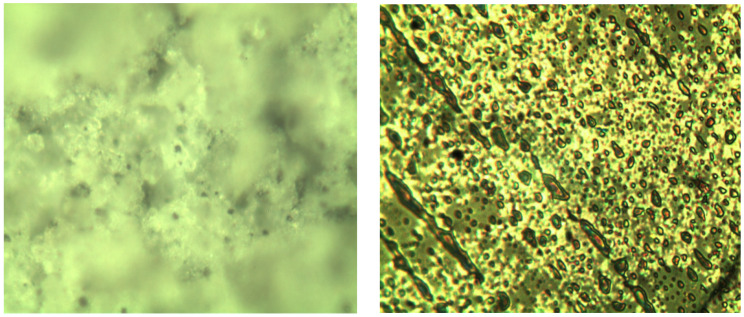
Resistance of samples ① and ② as a function of temperature in high-temperature water.

After incorporating and firing CaSO_4_, Ca(OH)_2_, MgSO_4_, and SiO_2_, the insulation resistance of specimen ② initially resembled that of specimen ①, with a rapid decrease in resistance. However, the rate of resistance change in specimen ② slowed compared to specimen ①. Over time, the resistance of specimen ② continued to decrease, though more gradually. After 72 h, its resistance remained in the megaohm range. Compared to specimen ①, specimen ② demonstrates considerably improved insulation performance and longevity. After the test specimen was removed, the surface was slightly duller compared to before the test, but the roughness was normal, and the color remained largely unchanged, still showing a light green hue (Figure 9). The thickness of the glaze surface was unchanged, and its microstructure appeared more compact. The improved insulation performance of specimen ② under high temperature and pressure in water is attributable to the addition of scale components, which may lead to the formation of an amorphous structure (Figure 10). This structural change enhances the material’s adhesion and insulation properties [27].

**Figure 11:**
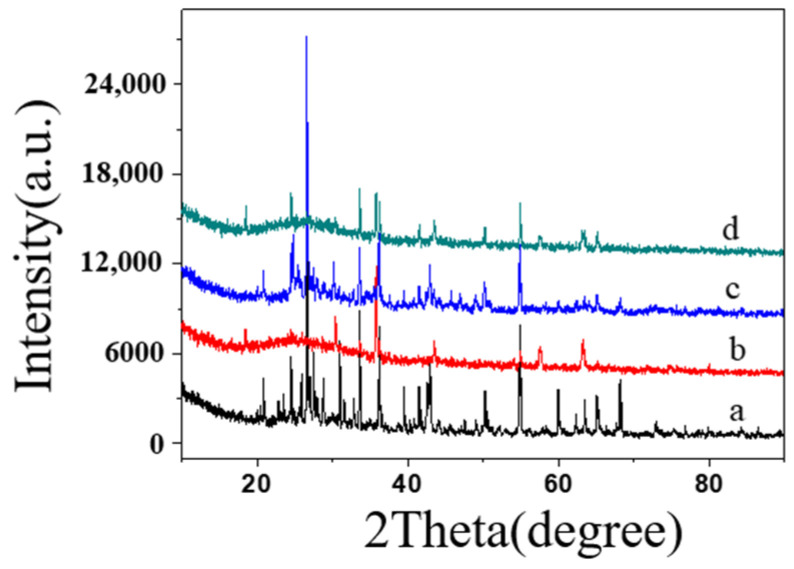
XRD spectra of firing specimen ① before firing specimen (**a**) and firing specimen after (**c**), specimen ② firing specimen before (**b**) and firing specimen after (**d**).

The samples were analyzed before and after the addition and calcination processes to better understand the impact of inorganic components on material properties. Figure 11 shows the XRD patterns of specimens ① (Figure 11a,c) and ② (Figure 11b,d) before and after firing. As observed, the patterns for specimens ① (Figure 11a) and ② (Figure 11b) before firing exhibited characteristic diffraction peaks at approximately 30.4°, 35.8°, 43.5°, 53.5°, 57.6°, and 63.3°, similar to those of pure SiO_2_-B. This result indicates no interaction between the various components before firing, with each existing as a separate molecule. The X-ray diffraction patterns of samples 1 and 2, fired at high temperatures, show broad, flat curves with a prominent peak at 2*θ* = 30°, characteristic of an amorphous state. The observed internal stress, which had insufficient time to dissipate during high-temperature firing and rapid cooling, formed an amorphous material. Despite this, the non-crystalline substance exhibits good compactness.

**Figure 12:**
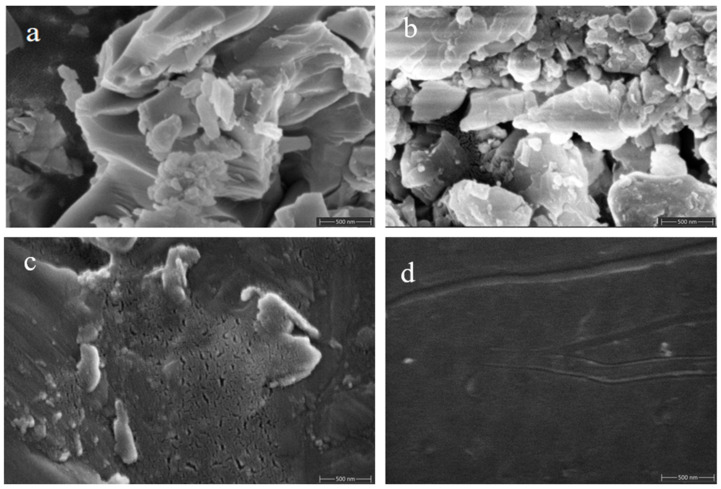
SEM images of specimens ① (**a**,**c**) and ② (**b**,**d**) before and after firing.

The surface morphology of specimens ① (Figure 12a,c) and ② (Figure 12b,d) before and after firing was recorded using SEM (Figure 12). A comparison of these images reveals that the unfired samples, both ① and ②, exhibit a mix of single crystal and polycrystalline structures with larger grains and noticeable grain boundaries, resulting in considerable surface roughness owing to gaps and cavities. After firing, both specimens have a smoother and more compact surface with improved gloss. This result indicates that the firing process transforms the samples from crystalline to amorphous, which aligns with the XRD results. However, sample ① exhibited defects such as cracking and fine holes after firing.

In contrast, sample ②, fired after incorporating scale components, displayed no surface defects such as holes, cracks, glaze shrinkage, or crystallization. This improvement is attributable to adding scale components, which inhibit crystallization and reduce the crystallization rate. This modification helps to mitigate glass glaze cracking, lower its thermal expansion coefficient, and enhance its thermal stability, which is crucial for resistance to high-temperature and high-pressure water conditions.

**Figure 13:**
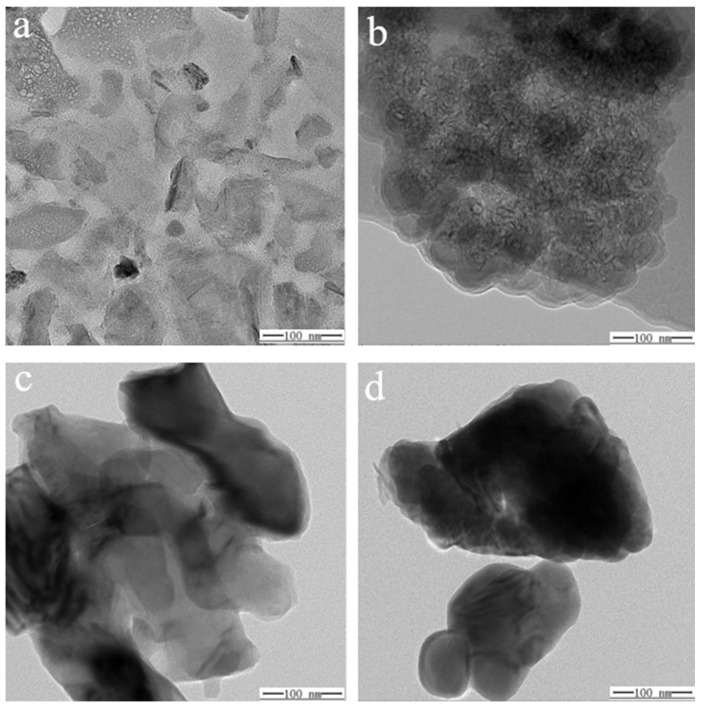
TEM of specimens ① (**a**,**c**) and ② (**b**,**d**) before and after firing.

**Figure 14:**
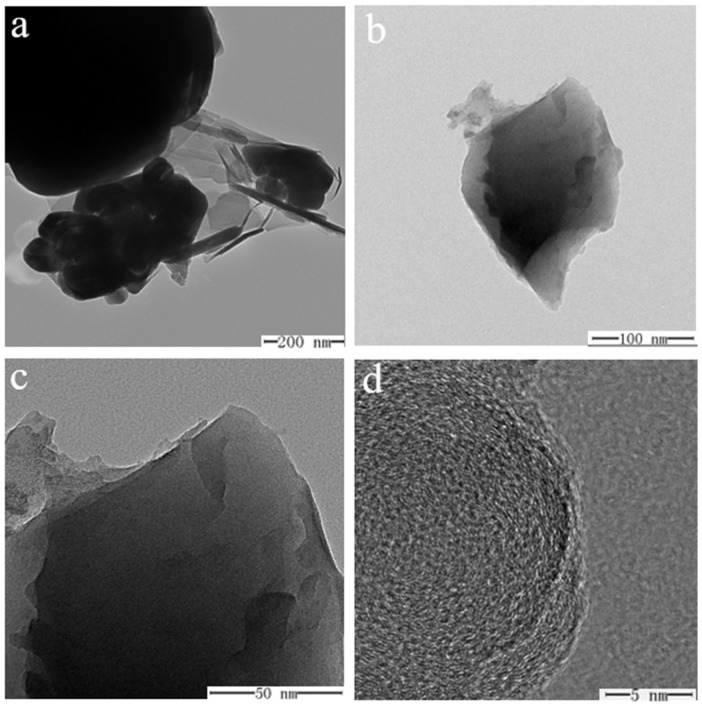
TEM images of specimen ② at different scales ((**a**) 200 nm, (**b**) 100 nm, (**c**) 50 nm, (**d**) 5 nm).

The internal morphology of specimens ① (Figure 13a,c) and ② (Figure 13b,d) before and after firing was recorded using TEM (Figure 13). The images reveal that the unfired structures of sample ① (Figure 13a) and sample ② (Figure 13b) are relatively fluffy, indicating that the components are individually distinct with weak bonding. After firing, the internal structure of both samples becomes denser, with sample ② (Figure 13c) showing a notably more compact arrangement, and (Figure 13d) adding the scale component in sample ② results in a more refined, flaky structure. Examining sample morphology ② at various scales (200, 100, 50, and 5 nm) (Figure 14a–d) reveals that the sample exhibits a flaky distribution after adding scale components. At a 5 nm scale (Figure 14d), the morphology of sample ② appears very dense with a distinct laminar distribution. This enhanced structure may result from the scale component acting as a cosolvent, promoting the formation of an amorphous state. Literature indicates that MgSO_4_ in glass glaze behaves similarly to CaSO_4_, providing free oxygen at high temperatures, effectively reducing the solution’s viscosity, and acting as a flux. It also inhibits the tendency for crystallization and reduces the crystallization rate. This result helps minimize cracking in the glass glaze, lowering its thermal expansion coefficient and improving its thermal stability. These properties are crucial for the material’s performance under high-temperature and high-pressure conditions in water.

**Figure 15:**
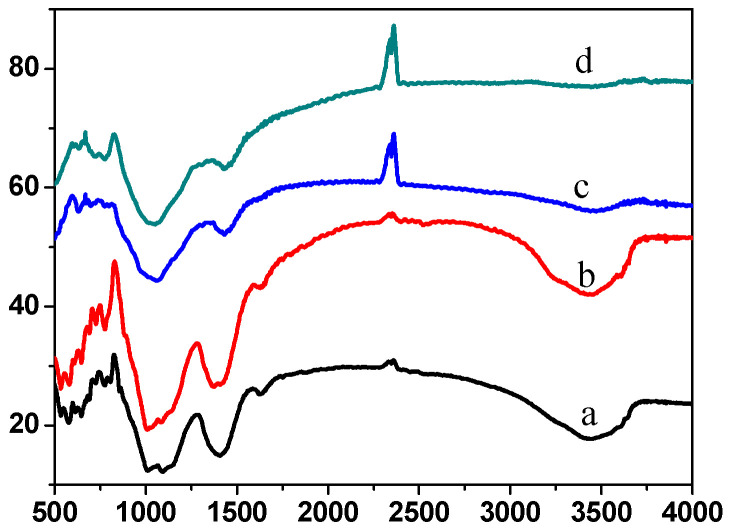
FT-IR spectra of before and after firing specimen ① before firing specimen (**a**) and firing specimen after (**c**), specimen ② firing specimen before (**b**) and firing specimen after (**d**).

For specimens ① and ② before and after firing, the spectra reveal distinct infrared absorption characteristics at 1011, 1092, 1407, 1625, 2336, 2360, and 3447 cm^−1^ (Figure 15). Notably, the peak at 3447 cm^−1^ represents the characteristic stretching vibration of OH^−^ groups, with high intensity and considerable broadening, indicating a high OH concentration before firing. The 2336 and 2360 cm^−1^ peaks correspond to the symmetric stretching vibration of CO_2_, likely present due to CO_2_ in the air. The absorption peak at 1636 cm^−1^ corresponds to the asymmetric stretching vibration of HCO_3_^−^, while the peak at 1407 cm^−1^ represents the symmetric stretching vibration of BO_3_^3−^, with a relatively high intensity. The absorption peaks at 1092 and 1011 cm^−1^ are characteristic of the symmetric stretching vibration of CO_3_^2−^, and the peak at 826 cm^−1^ is attributed to the asymmetric stretching vibration of BO_3_^3−^. These observations suggest that the raw material before firing does not contain considerable amounts of OH^−^, HCO_3_^−^, or CO_3_^2−^ groups. The infrared spectrum of fired specimens ① and ② mainly shows peaks at 1060, 1434, 2336, and 2360 cm^−1^ (Figure 15a,b). The disappearance of 1625 and 3447 cm^−1^ peaks indicates a reduction in HCO_3_^−^ and OH^−^. The peak at 826 cm^−1^ corresponds to the asymmetric stretching vibration of BO_3_^3−^, while the peaks at 1060 and 1434 cm^−1^ are characteristic of the symmetric stretching vibrations of CO_3_^2−^ and BO_3_^3−^, respectively. These peaks have shifted to lower wavelengths than those observed before firing. This shift indicates that new bonding interactions between carbonate and borate likely develop during firing, contributing to the special glass glaze system’s stability and resistance to high-temperature and high-pressure water. This finding aligns with the SEM results. Adding inorganic components facilitates the formation of a non-static state, resulting in a smoother surface, improved adhesion, and enhanced durability.

### 3.3. Test in Water under Variable Temperature and Pressure

Table 8 summarizes the performance of test specimens ① and ② in water under varying temperature and pressure conditions. Because the test specimens are intended for use in environments exceeding 200 °C, testing began at temperatures above 150 °C. During the test, the pressure in the container gradually increased. Once reaching 12 MPa, the pressure regulating valve was adjusted to maintain a 12 ± 0.5 MPa pressure during heating.

**Figure 16:**
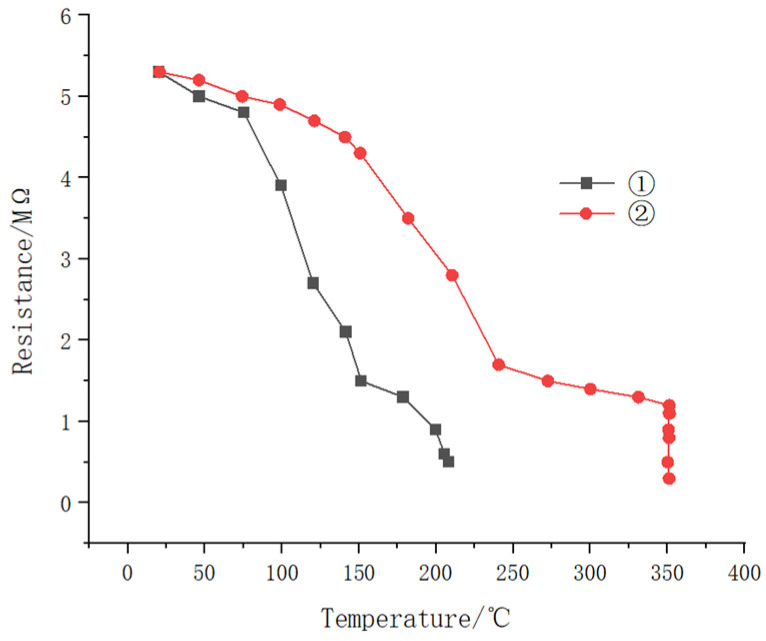
The resistance of sample ① and sample ② varies with temperature in high temperature water.

As shown in Figure 16, the resistance of both samples decreased significantly with increasing temperature. At similar temperatures, sample ① exhibited lower resistance than that of sample ②, indicating that sample ① had considerably different insulation performance compared to that of sample ②. Additionally, sample ① experiences insulation failure at temperatures below 240 °C, characterized by very low resistance, while sample ② maintains its resistance up to 350 °C, demonstrating superior high-temperature performance.

Comparing the data in Table 6, test specimen ①, when continuously heated and pressurized to 200 °C in water, showed a decrease in insulation resistance to 0.6 Ω in just 0.5 h, indicating that the insulating layer was compromised. Upon removal, it was observed that a small portion of the material had detached, with a falling radius of approximately 0.4 mm. The surface appeared rough and tarnished, with a light white powder adhering to it. In contrast, although the insulation resistance of test specimen ② gradually decreased with continued heating and pressure, it retained strong insulating properties after 72 h. After removing test specimen ②, a layer of pale white scale powder was found on the surface. Once the scale was removed, the glaze appeared slightly duller, and the surface roughness had increased compared to before the test. However, there was no noticeable discoloration in the water.

These results show that adding CaSO_4_, MgSO_4_, Ca(OH)_2_, and SiO_2_ to the glass glaze considerably enhances insulation performance, temperature resistance, and pressure resistance. The material has been verified against the conditions and requirements of heavy oil thermal recovery sites and is confirmed to meet all on-site usage requirements.

A temperature shock test further assessed the insulation performance of the material under high temperature and high pressure in water, with experimental data presented in Table 9. Test specimen ② was subjected to five cycles of temperature shocks, alternating between 500 °C air and 20 °C water, without any surface detachment. As shown in Table 6, the insulation performance in water remains largely unchanged, demonstrating robust insulation capabilities.

To further evaluate how the addition of inorganic components affects the resistance of special glass glaze to high-temperature and high-pressure water, experiments with individual additions of CaSO_4_ (sample ③), MgSO_4_ (sample ④), and Ca(OH)_2_ (sample ⑤) to the special glass glaze were performed. The results indicate that these samples, with inorganic components added alone, show poor stability after firing, similar to sample ①. While they exhibit some resistance to high-temperature and high-pressure water, their stability is inadequate. Thus, incorporating inorganic components (CaSO_4_, MgSO_4_, and Ca(OH)_2_) is crucial for achieving long-term resistance under such conditions.

## 4. Conclusions

A new composite material exhibits insulating properties under high temperatures and high pressure in water. This material is created by incorporating inorganic components—CaSO_4_, Ca(OH)_2_, MgSO_4_, and SiO_2_—into a special glass glaze. The primary components of the composite are SiO_2_, BaO, CaO, ZnO, B_2_O_3_, TiO_2_, Fe_2_O_3_, SO_3_, Cr_2_O_3_, clay, CaSO_4_, Ca(OH)_2_, MgSO_4_, and SiO_2_. The composite was applied to 316L stainless steel glass using a specialized firing process, resulting in an average sintered thickness of 0.1 mm and a surface roughness of 0.05 mm. The initial and composite glaze layers were evaluated for performance under high temperatures and high pressure in water. Composites doped with inorganic components can sustain their insulating properties for up to 72 h in high-temperature and high-pressure water. In addition, they maintain resistance in the megaohm range for several hours under these conditions, with no functional damage observed. This new insulating material has been developed into a sensor for measuring water holdup in horizontal wells under high-temperature and high-pressure water environments.

## Figures and Tables

**Table 1:** The main composition and content of the underglaze with special glass as the main material.

Chemical Formula	SiO_2_	BaO	CaO	ZnO	B_2_O_3_	TiO_2_	Fe_2_O_3_	SO_3_	Cr_2_O_3_	Clay
Component content %	27.1505	27.7305	2.607	2.9805	4.1339	2.0336	0.012	0.014	28.504	4.834

**Table 2:** Chemical composition and proportion.

Chemical formula	SiO_2_	BaO	CaO	ZnO	B_2_O_3_	TiO_2_	Fe_2_O_3_
Component content %	25.025	17.016	4.6	1.829	2.537	1.248	0.007
Chemical formula	SO_3_	Cr_2_O_3_	Clay	CaSO_4_	MgSO_4_	Ca(OH)_2_	
Component content %	0.009	17.491	2.966	6.818	6.818	13.636	

**Table 3:** Chemical composition and proportion of specimen ③.

Chemical formula	SiO_2_	BaO	CaO	ZnO	B_2_O_3_	TiO_2_	Fe_2_O_3_
Component content %	25.025	17.016	4.6	1.829	2.537	1.248	0.007
Chemical formula	SO_3_	Cr_2_O_3_	Clay	CaSO_4_	MgSO_4_	Ca(OH)_2_	
Component content %	0.009	17.491	2.966	27.272	0.000	0.000	

**Table 4:** Chemical composition and proportion of specimen ④.

Chemical formula	SiO_2_	BaO	CaO	ZnO	B_2_O_3_	TiO_2_	Fe_2_O_3_
Component content %	25.025	17.016	4.6	1.829	2.537	1.248	0.007
Chemical formula	SO_3_	Cr_2_O_3_	Clay	CaSO_4_	MgSO_4_	Ca(OH)_2_	
Component content %	0.009	17.491	2.966	0.000	27.272	0.000	

**Table 5:** Chemical composition and proportion of specimen ⑤.

Chemical formula	SiO_2_	BaO	CaO	ZnO	B_2_O_3_	TiO_2_	Fe_2_O_3_
Component content %	25.025	17.016	4.6	1.829	2.537	1.248	0.007
Chemical formula	SO_3_	Cr_2_O_3_	Clay	CaSO_4_	MgSO_4_	Ca(OH)_2_	
Component content %	0.009	17.491	2.966	0.000	0.000	27.272	

**Table 6:** Relationship between test temperature and resistance of the composite glaze in air.

Specimen	Temperature /°C	Resistance /MΩ	Specimen	Temperature /°C	Resistance /MΩ
①	17.2	∞	②	17.1	∞
	50.5	∞		50.3	∞
	82.3	∞		81.2	∞
	109.6	∞		109.6	∞
	142.3	∞		142.1	∞
	171.8	∞		171.1	∞
	198.2	∞		199.8	∞
	233.5	980.3		228.9	∞
	258.6	925.5		259.7	985.6
	288.8	880.3		291.2	936.9
	324.1	830.8		319.8	893.6
	348.7	760.5		351.5	847.5
	381.3	725.3		379.8	815.3
	411.5	663.7		411.2	765.4
	448.3	567.9		449.5	702.3

**Table 7:** Relation between the resistance of the material in room-temperature water and test duration.

Specimen	Temperature/°C	Time/h	Resistance /MΩ	Specimen	Temperature/°C	Time/h	Resistance /MΩ	Specimen	Temperature/°C	Time/h	Resistance /MΩ
①	20	0	98.5	②	20.5	0	∞	②	19.3	36	3.9
	18.9	0.5	5.5		21.0	0.5	99.6		19.2	39	3.8
	17.4	3	4.8		21.2	3	16.6		19.4	42	2.6
	21.3	6	3.5		22.4	6	15.4		19.6	45	2.5
	19.5	9	3.2		23.6	9	15.1		20.4	48	2.3
	19.7	12	2.6		23.1	12	12.4		20.6	51	2.2
	20.2	15	2.1		22.6	15	10.9		19.5	54	1.9
	20.6	18	1.4		22.5	18	9.8		18.6	57	1.7
	18.9	21	0.5		21.4	21	8.6		18.4	60	1.5
	18.8	24	0.3		19.6	24	7.6		18.3	63	1.5
	18.0	27	0.1		19.1	27	7.2		18.1	66	1.3
	17.9	30	0.6 × 10^−6^		18.5	30	5.3		17.0	69	1.3
	18.2	33	0.5 × 10^−6^		18.2	33	4.2		17.2	72	0.8

**Table 8:** Relationship between temperature and resistance in water over time.

Specimen	Temperature	Water Pressure	Time	Resistance	Specimen	Temperature	Water Pressure	Time	Resistance	Specimen	Temperature	Water Pressure	Time	Resistance
	/°C	/MPa	/h	/MΩ		/°C	/MPa	/h	/MΩ		/°C	/MPa	/h	/MΩ
①	20.2	0.10	1.1	5.3	②	20.5	0.10	1.1	5.3	②	331.3	12.05	20.0	1.3
	46.4	0.10	1.5	5.0		46.2	0.10	1.5	5.2		351.3	12.00	24.0	1.2
	75.3	0.10	1.8	4.8		74.2	0.10	1.8	5.0		351.5	12.00	28.0	1.1
	99.4	0.10	2.1	3.9		98.6	0.10	2.1	4.9		351.3	12.00	34.0	1.1
	120.3	0.21	2.6	2.7		121.0	0.21	2.6	4.7		351.5	12.00	40.0	1.1
	141.5	0.42	3.0	2.1		140.9	0.42	3.0	4.5		351.2	12.00	46.0	1.1
	151.5	0.49	3.5	1.5		150.8	0.49	3.5	4.3		350.8	12.00	52.0	0.9
	178.7	0.98	5.5	1.3		181.9	1.09	6.0	3.5		351.2	12.00	60.0	0.8
	199.6	1.58	7.5	0.9		210.5	1.98	9.0	2.8		350.3	12.00	65.0	0.5
	205.6	1.75	8.0	0.6		240.6	3.31	12.0	1.7		351.2	12.00	72.0	0.3
	208.3	1.81	9.0	0.5		272.5	14.5	14.5	1.5		351.1	12.00	73.2	0.3 × 10^−6^
	239.2	3.32	12.0	0.5 × 10^−6^		300.2	8.93	17.0	1.4					

**Table 9:** Specimen ② Insulation performance in normal temperature water after shock from sudden temperature change.

Temperature /°C	Time/h	Resistance /MΩ	Temperature /°C	Time/h	Resistance /MΩ
21.5	0	88.5	21.3	40	2.6
21.5	5	15.1	21.2	45	2.5
21.6	10	14.0	20.8	50	2.0
22.0	15	10.2	21.0	55	1.8
22.3	20	8.5	21.4	60	1.4
22.8	25	7.4	21.1	65	1.3
22.4	30	4.8	21.0	70	0.9
22.4	35	3.7	20.9	75	0.8

## Data Availability

Data are contained within the article.
